# Complete Genome Sequences of Two Isolates of Fusobacterium necrophorum subsp. funduliforme, Obtained from Blood from Patients with Lemierre’s Syndrome

**DOI:** 10.1128/MRA.01577-18

**Published:** 2019-01-24

**Authors:** Catrine Lyster, Lena Hagelskjær Kristensen, Jørgen Prag, Anders Jensen

**Affiliations:** a Department of Biomedicine, Aarhus University, Aarhus, Denmark; b Department of Medicine, Viborg Regional Hospital, Viborg, Denmark; c Department of Clinical Microbiology, Viborg Regional Hospital, Viborg, Denmark; Broad Institute of MIT and Harvard University

## Abstract

Two isolates (F1260 and F1291) of Fusobacterium necrophorum subsp. funduliforme were recovered from blood from patients with Lemierre’s syndrome. Here, we report the complete genome sequences of these two isolates. The genomes of F1260 and F1291 comprise one chromosome with lengths of 2.29 and 2.14 Mb, respectively.

## ANNOUNCEMENT

Fusobacterium necrophorum is a Gram-negative anaerobic bacterium and is the etiological agent of Lemierre’s syndrome, a rare life-threatening disease that mostly originates from the throat and affects mainly teenagers and young adults (1, 2). F. necrophorum has also been associated with tonsillitis and peritonsillar abscesses (3, 4). F. necrophorum is divided into two subspecies, F. necrophorum subsp. necrophorum and F. necrophorum subsp. funduliforme, with F. necrophorum subsp. necrophorum being an animal pathogen (5, 6).

Here, we present the complete genome sequences of two F. necrophorum subsp. funduliforme isolates, F1291 and F1260. Both isolates were recovered from blood from Lemierre’s syndrome patients. The two isolates were previously sequenced with Illumina short-read sequencing, which resulted in 37 contigs for isolate F1291 (GenBank accession no. LVEU00000000) and 62 contigs for isolate F1260 (GenBank accession no. LVDZ00000000). The goal of this project was to generate complete closed genomes that can serve as reference genomes.

Genomic DNA was isolated directly from bacterial colonies grown on 5% blood agar medium (SSI, Denmark) at 37°C for 48 h under anaerobic conditions using the MasterPure DNA purification kit (Epicentre). Genomic libraries were constructed using the PacBio DNA template prep kit version 3.0 according to the manufacturer’s instructions, and the resulting libraries were sequenced using a single-molecule real-time (SMRT) sequencing cell on a PacBio RS II machine at GATC (Germany). After the raw reads were filtered, 75,825 reads, with an *N*_50_ read length of 15,167 bp and a mean length of 9,807 bp, and 75,945 reads, with an *N*_50_ read length of 18,600 bp and a mean length of 11,625 bp, were retained for isolates F1291 and F1260, respectively. The assemblies were done with HGAP3 (7) and resulted in a single contig for both isolates, with average 281-fold and 300-fold coverages for F1291 and F1260, respectively. These contigs corresponded to the circular chromosome of the two isolates, indicated by the sequence overlap at the ends of both contigs, and had lengths of 2,288,480 bp (G+C content, 35.2%) and 2,135,983 bp (G+C content, 35.3%) for isolates F1260 and F1291, respectively.

Gene prediction and annotation were performed using RAST (8). RAST predicted 2,324 and 2,065 genes on the circular chromosomes for F1260 and F1291, respectively. The larger genome size and number of predicted genes in F1260 were mostly due to stretches of phage-associated genes and mobile elements, as determined by the annotation of genes done by RAST, in which most of the genes had an unknown function. Alignment of the two genomes by Mauve (9) showed complete synteny, except for two small inverted regions. One region consisted of three genes, with two of the genes being identical at the protein level and identified as outer membrane proteins/porins. The gene had 56% identity to the FomA adhesion protein sequence of Fusobacterium nucleatum. The importance of the duplication of this gene in the adhesion and colonization process of F. necrophorum has yet to be determined. Genes for known putative virulence factors in F. necrophorum were found in both isolates and included genes for hemolysin, leukotoxin, hemin receptor, ecocin, and filamentous hemagglutinin.

The complete genomes of human isolates of F. necrophorum subsp. funduliforme may contribute to our understanding of the pathogenesis of invasive F. necrophorum infections in humans, such as Lemierre’s syndrome.

### Data availability.

The genome sequences of isolates F1260 and F1291 have been deposited in GenBank under the accession no. CP019306 and CP018196, respectively. The raw reads have been deposited in the NCBI SRA database under the accession no. SRR8271517 and SRR8281305 for isolates F1260 and F1291, respectively.
